# *Loa loa* and *Mansonella perstans* microfilaremia in the department of Lékoumou, Republic of Congo

**DOI:** 10.1186/s13071-023-06056-w

**Published:** 2023-12-09

**Authors:** Marlhand C. Hemilembolo, Jérémy T. Campillo, Ange Clauvel Niama, Sébastien D. S. Pion, François Missamou, Michel Boussinesq, Richard R. Bileckot, Cédric B. Chesnais

**Affiliations:** 1 TransVIHMI, Université de Montpellier, Institut de Recherche pour le Développement (IRD), INSERM Unité 1175, Montpellier, France; 2 Programme National de Lutte Contre l’Onchocercose, Ministère de la Santé et de la Population, Brazzaville, République du Congo; 3 Faculté des Sciences de la santé de l’Université Marien NGOUABI, Brazzaville, République du Congo

**Keywords:** Filariasis, *Loa loa*, *Mansonella perstans*, Parasitological survey, Republic of Congo

## Abstract

**Background:**

Loiasis is endemic in the northern and western part of the Republic of Congo. Between 2004 and 2010, surveys were conducted, using the RAPLOA method, in all departments of the Republic of Congo to assess the distribution of loiasis. Prior to 2004, only two parasitological surveys on loiasis had been conducted in Congo and mainly in the Department of Lékoumou, in the southwestern of the country. In 2019, we conducted a parasitological survey in this same department, more than 30 years after the first surveys.

**Methods:**

The study was conducted in 21 villages. *Loa loa* and *Mansonella perstans* microfilaremia levels were quantified using 50 µl calibrated blood smears.

**Results:**

A total of 2444 individuals were examined. The median age of the screened individuals was 43 (interquartile range: 30–57, range: 18–91) years old. The overall prevalences of *L. loa* and *M. perstans* microfilaremia were 20.0% [95% confidence intervals (CI) 18.0–21.6%] and 1.0% (95% CI 0.6–1.4%) respectively. The proportion of individuals with a microfilarial density of *L. loa* > 8000 mf/ml and > 30,000 mf/ml were 5.1% (95% CI 4.3–6.1%) and 1.1% (95% CI 0.8–1.7%), respectively. The overall community microfilarial load was 3.4 mf/ml.

**Conclusions:**

Prevalences and intensities of *L. loa* infection remained generally stable between the late 1980s and 2019 in the Lékoumou Department. In contrast, parasitological indicators for *M. perstans* have declined sharply in the intervening years for an unknown reason.

**Graphical Abstract:**

**Figure:**
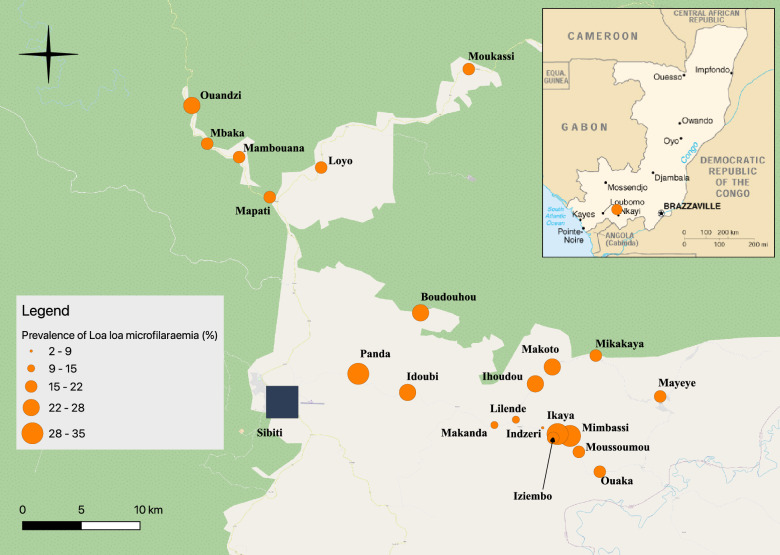

**Supplementary Information:**

The online version contains supplementary material available at 10.1186/s13071-023-06056-w.

## Background

Loiasis is a parasitic infection resulting from the presence of the filarial worm *Loa loa*, transmitted between humans by tabanid vectors, primarily *Chrysops silacea* and *C. dimidiata* and mostly endemic in the forested regions of Central Africa [1]. An estimated 15 million people live in areas at high risk of infection [2]. Surveys conducted in the Republic of Congo have revealed the endemicity of loiasis in the western and northern regions of the country [2–5]. In the Lékoumou Department, the initial assessment of *L. loa* microfilaremia prevalence took place between 1985 and 1989, revealing an estimated 18.9% prevalence across all seven villages. The median individual microfilarial densities (MFDs) varied by village: from 800 to 3100 microfilariae per milliliter of blood (mf/ml) for the Bantu population and from 150 to 2250 mf/ml for the Pygmies [4]. A subsequent study involving adult Bantu from two villages in the same region indicated microfilaremia prevalences of 27.7 and 29.5% [3]. Furthermore, a survey conducted in 2004 revealed *Loa loa* microfilaremia prevalences ranging from 7.5 to 50.8% across 15 villages [6].

In 2019, we conducted an additional parasitological survey across 21 villages of the Lékoumou Department, including four villages surveyed in 1989. This survey not only provides an updated perspective on the distribution of loiasis in the department but also allows an evaluation of its endemicity evolution over a 30-year span. Additionally, we assessed the prevalence of *Mansonella perstans* microfilaremia. Given the hypoendemic nature of onchocerciasis in the Lékoumou Department, it is a potential target for future onchocerciasis elimination activities. Consequently, we present epidemiological indicators identifying the population at risk of *L. loa*-related post-ivermectin serious adverse events.

## Methods

The survey was conducted as part of a screening phase for a randomized controlled trial assessing the safety and efficacy of levamisole in individuals with *L. loa* microfilaremia [7]. To promptly address potential adverse events during the trial, screening was restricted to villages within a 1-h drive from Sibiti, continuing until the desired sample size was attained. In these villages, all volunteers aged ≥ 18 years were then invited to the screening, which took place in October 2019 across 21 villages.

The Lékoumou Department encompasses an expanse of 20,950 km^2^ and, as of 2018, was home to 135,643 inhabitants [8]. Based on routine information from the long-acting insecticide-treated net distribution program, the collective population of the 21 villages involved in this study is reported as 20,651. The region is predominantly characterized by forested surroundings, and the primary activities of the population include food crop farming and arboriculture [8].

An informational document, accessible in French, Kituba and Lingala (two national languages), outlining the objectives and procedures of the survey was distributed to the population prior its commencement. Potential participants were assured that their identity and test results would be treated with utmost confidentiality. Each individual who agreed to take part in the survey formally signed an informed consent form. The study received approval from the Ethics Committee for Research in Health Sciences of the Republic of Congo (no. 226/MRSIT/IRSSA/CERSSA), and an administrative authorization (no. 469/MSP/CAB/UCPP-19) was granted by the Ministry of Health and Population.

Following the registration of participants’ names, sexes and ages, a blood sample was collected through a finger prick between 10 a.m. and 4 p.m. Using a sterile, single-use lancet and a non-heparinized capillary tube, a calibrated 50-µl-thick smear was prepared for each subject. Within 24 h, dehemoglobinization occurred. Subsequently, each thick smear was stained with Giemsa and thoroughly examined under a microscope to identify and quantify *L. loa* and *M. perstans* microfilariae (mf). Individual microfilarial densities (MFDs) were expressed as mf/ml.

The prevalence and intensity of *L. loa* and *M. perstans* microfilaremia were analyzed by sex and age groups aligned with the seven quantiles (18–23, 24–32, 33–40, 41–47, 48–55, 56–65 and 66–91 years). The 95% confidence intervals (95% CI) for each prevalence were computed using Wilson's method (uncorrected for continuity) [9]. Mean MFDs were expressed as the arithmetic mean of individual MFDs in the entire study population, encompassing both microfilaremic and amicrofilaremic individuals, and as the geometric mean of MFDs specifically within microfilaremic individuals [10, 11]. The mean MFDs in each sex were compared using Student's *t*-test.

Proportions of individuals with *L. loa* MFD > 8000 mf/ml and 30,000 mf/ml were computed. This calculation is significant because individuals with MFD > 8000 mf/ml have an elevated risk of experiencing marked or severe adverse reactions, while those with > 30,000 mf/ml are at an increased risk of developing severe adverse reactions, including neurological effects, following the use of ivermectin [12]. Finally, for each village, the *L. loa* Community Microfilarial Load (CMFL) was calculated [13] using the following formula: ${\text{e}}^{\sum^\frac{\ln (x + 1)}{n}} - 1$ [14], where x is the individual MFD and n is the number of individuals examined (CMFLs were calculated on the entire study population).

The relationship between prevalence and each indicator *L. loa* infection intensity in this study was assessed using Spearman's correlation coefficient (*r*). Subsequently, we employed an unmatched proportion comparison test to compare prevalence between 2004 and 2019 (or, in the case of Panda, between the 1985–1989 period and 2019). Additionally, Student’s test for unmatched data was applied to compare CMFL between the two periods. Data were analyzed using R software, version 4.2.1.

## Results

**Fig. 1:**
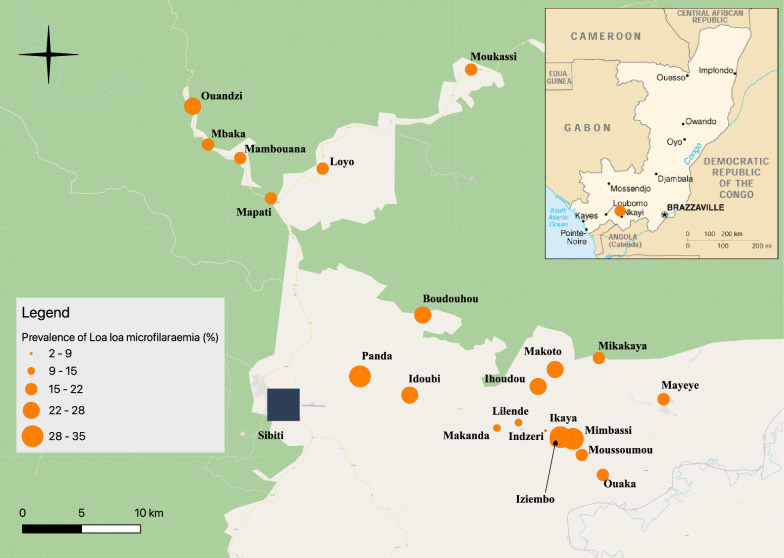
Distribution of *Loa loa* microfilaremia prevalence by surveyed village in 2019

A total of 2444 individuals, ranging from 18 to 91 (median: 43) years old participated in the survey. Among the participants, males accounted for 51.6% (*n* = 1262). *Loa loa* mf prevalence exhibited variation across villages, ranging from 2.8 to 34.9% (Table 1). Notably, 13 out of the 21 villages reported prevalence values surpassing 20%, while two villages recorded prevalence exceeding 30%. In populated areas like Mayéyé, Mapati and Loyo, neighboring communities may exhibit divergent prevalence values of *L. loa* mf, as illustrated in Fig. 1.

**Table 1:** Geographical coordinates, total population, number of subjects examined, number of microfilaremic subjects for *Loa loa* and *Mansonella perstans*, prevalence of *L. loa* and *M. perstans* microfilaremia and indicators of intensity of *L. loa* microfilaremia in the 21 villages surveyed

Village	Longitude	Latitude	Pop.	Ex	AgeM (IQR)	N. *Loa* +	Prev. mf *Loa* (IC 95%)	CMFL *Loa*	MA *Loa*	% > 8000 mf/ml	% > 30,000 mf/ml	N. *Mp* +	Prev mf *Mp* (IC 95%)
Boudouhou	− 3.617431	13.457584	1323	113	43 (32–56)	25	22.1 (14.3–32.7)	4.0	1526	5.3	0.9	0	0
Idoubi	− 3.678527	13.44763	535	56	51 (43–69)	13	23.2 (12.4–39.7)	4.7	933	3.6	0.0	2	3.6 (0.4–12.9)
Ihoudou	− 3.671935	13.545637	520	69	40 (27–56)	17	24.6 ((14.6–39.4)	5.1	1344	7.2	0.0	0	0
Ikaya	− 3.711913	13.572193	423	63	52 (36–64)	22	34.9 (21.9–52.9)	12.6	2453	9.5	1.6	1	1.6
Indzeri	− 3.705659	13.551219	390	36	44 (24–56)	1	2.8 (0.1–15.5)	0.4	2572	2.8	2.8	0	0
Iziembo	− 3.709723	13.560436	530	33	47 (32–59)	7	21.2 (8.5–43.7)	2.8	1181	3.0	3.0	0	0
Lilende	− 3.699444	13.530688	427	69	45 (34–62)	10	14.5 ((6.9–26.7)	1.8	389	0.0	0.0	1	1.4 (0.03–8.1)
Loyo	− 3.05896	13.381352	2085	248	43 (29–56)	43	17.3 (12.5–23.4)	2.5	1360	4.8	1.6	3	1.2 (0.2–3.5)
Makanda	− 3.703558	13.514197	632	104	45 (32–57)	11	10.6 (5.3–18.9)	1.3	910	2.9	1.0	0	0
Makoto	− 3.658986	13.558613	600	87	44 (30–57)	20	23.0 (14.0–35.5)	4.2	877	3.4	0.0	1	1.1 (0.02–6.4)
Mambouana	− 3.497895	13.318404	1375	71	43 (28–57)	15	21.1 (11.8–34.8)	2.9	290	0.0	0.0	1	1.4 (0.03–7.8)
Mapati	− 3.528635	13.341885	1342	100	45 (30–60)	17	17.0 (9.9–27.2)	2.4	1144	3.0	2.0	1	1.0 (0.03–5.6)
Mayeye	− 3.681605	13.641355	3647	484	43 (31–55)	82	16.9 (13.5–21.0)	2.7	1632	4.1	1.0	9	1.9 (0.9–3.5)
Mbaka	− 3.487612	13.293929	390	81	39 (30–50)	17	21.0 (12.2–33.6)	4.9	2392	9.9	1.2	0	0
Mikakaya	− 3.650241	13.59198	1180	148	43 (30–57)	29	19.6 (13.1–28.1)	3.1	1053	4.1	0.0	1	0.7 (0.01–3.8)
Mimbassi	− 3.71051	13.562675	684	56	52 (39–64)	17	30.4 (17.7–48.6)	9.0	1852	8.9	1.8	0	0
Moukassi	− 3.430349	13.494546	1328	141	46 (35–58)	30	21.3 (14.4–30.4)	3.1	675	3.5	0.0	1	0.7 (0.02–4.0)
Moussoumou	− 3.72416	13.578987	787	90	40 (28–63)	15	16.7 (9.3–27.5)	2.2	586	3.3	0.0	0	0
Ouaka	− 3.739396	13.595035	1528	236	39 (27–55)	50	21.2 (15.7–27.9)	4.6	2793	9.7	3.0	1	0.4 (0.01–2.4)
Ouandzi	− 3.458303	13.282144	401	87	39 (28–53)	21	24.1 (14.9–36.9)	5.3	2183	8.0	2.3	0	0
Panda	− 3.664232	13.409826	524	72	47 (29–62)	21	29.2 (18.1–44.6)	7.8	1680	6.9	1.4	0	0
Total			20,651	2444	43 (30–57)	483	20.0 (18.1–21.6)	3.4	1487	5.1	1.1	22	1.0 (0.6–1.4)

Pop., total village population; Ex, number of subjects examined; AgeM, median age of subjects examined; IQR, interquartile range; N. *Loa* + , number of microfilaremic subjects for *Loa*; Prev. mf *Loa*, prevalence of microfilaremia in *Loa*; CI 95%, 95% confidence interval; CMFL *Loa*, community microfilaremic load for *L. loa*; MA *Loa*, arithmetic mean of individual *L. loa* microfilarial densities; % > 8000 mf/ml, percentage of subjects with > 8000 *L. loa* mircofilariae per ml blood; % > 30,000 mf/ml, percentage of subjects with > 8000 *L. loa* microfilariae per ml blood; N Mp + , number of subjects microfilaremic for *Mansonella perstans*; Prev. mf Mp, prevalence of microfilaremia for *M. perstans*

The prevalence of *L. loa* microfilaremia was found to be twice as high in men (25.6%, 95% CI 22.9–28.5) compared to women (12.7%, 95% CI 10.7–14.9). Additionally, this prevalence showed a progressive increase with age, starting from 10.9% (95% CI 7.7–14.9) in individuals aged 18–23 years to 27.3% (95% CI 21.7–33.8) in those aged 66–91 years (Table 2).

**Table 2:** Prevalence of *Loa loa* microfilaremia and geometric MFD mean by age group and sex

Age	Men					Women					All				
	N	N +	Prevalence (IC 95%)	GM	MFD median (range)	N	N +	Prevalence (IC 95%)	GM	MFD median (range)	N	N +	Prevalence (IC 95%)	GM	MFD median (range)
18–23	216	28	13.0 (8.6–18.7)	836	1350 (20–92600)	142	11	7.7 (3.9–13.9)	897	700 (40–16900)	358	39	10.9 (7.7–14.9)	853	1240 (20–92600)
24–32	191	42	22.0 (15.8–29.7)	2084	3580 (20–35000)	173	10	5.8 (2.8–10.6)	915	1450 (20–19400)	364	52	14.3 (10.7–18.7)	1779	3360 (20–35000)
33–40	207	57	27.5 (20.9–35.7)	2196	3100 (40–62700)	140	16	11.4 (6.5–18.6)	3275	3620 (200–33840)	347	73	21.0 (16.5–26.5)	2397	3200 (40–62700)
41–47	178	41	23.0 (16.5–31.2)	1653	2460 (20–41800	152	12	7.9 (4.1–13.8)	4068	3800 (800–54660)	330	53	16.1 (12.0–21.0)	2027	2460 (20–54660)
48–55	175	59	33.7 (25.7–43.5)	1933	2000 (20–109000)	189	19	15.3 (6.1–15.7)	1547	4000 (20–72080)	364	88	24.2 (19.4–29.8)	1796	2120 (20–109000)
56–65	161	51	31.7 (23.6–41.6)	1634	1800 (20–57060)	216	44	20.4 (14.8–27.3)	1884	2040 (20–70600)	377	95	25.2 (20.4–30.8)	1746	1980 (20–70600)
66–91	134	45	33.6 (24.5–44.9)	1230	900 (40–91600)	170	38	22.4 (15.8–30.7)	1753	1550 (60–62000)	304	83	27.3 (21.7–33.8)	1447	1400 (40–91600)
TOTAL	1262	323	25.6 (22.9–28.5)	1664	2140 (20–109000)	1182	150	12.7 (10.7–14.9)	1818	2070 (20–72080)	2444	483	20.0 (18.0–21.6)	1714	2100 (20–109000)

N, number of subjects examined; N +  = number of subjects with *L. loa* microfilaremia; GM, geometric mean (among individuals with *L. loa* MFD); MFD, microfilarial density

For *M. perstans*, only 22 out of 2444 individuals (1.0%, 95% CI 0.6–1.4) had *M. perstans* mfs (refer to Additional file 1: Fig. S1). The prevalence of microfilaremia ranged from 0 to 3.6% across villages (Table 1) with an overall CMFL of 0.01 mf/ml.

*The L. loa* MFD ranged from 20 to 109,000 mf/ml, with an arithmetic mean of 1487 mf/ml (95% CI 1485–1489) in the total population. It was significantly higher in men (1984 mf/ml) than in women (957 mf/ml) (*t*-test, *t*_(2442)_ = − 3.7285, *P* < 0.001).

The proportions of individuals with MFD ˃ 8000 mf/ml and those with MFD ˃ 30,000 mf/ml were 5.1% (95% CI 4.3–6.1) and 1.1% (95% CI 0.8–1.7), respectively, among all examined individuals. The overall CMFL across the 21 villages was 3.4 mf/ml, and detailed results for each village are available in Table 1.

At the community level, the prevalence of *L. loa* exhibited a positive and significant correlation with CMFL (Spearman’s correlation coefficient, *r*_s_ = 0.9, 95% CI 0.8–1.0, *P* < 0.001), with the proportion of individuals with MFD ˃ 8000 mf/ml (Spearman’s correlation coefficient, *r*_s_ = 0.6, 95% CI 0.2–0.8, *P* = 0.004) and with the arithmetic mean MFD (Spearman’s correlation coefficient, *r*_s_ = 0.5, 95% CI 0.1–0.7, *P* = 0.039). However, no significant correlation was observed with the proportion of individuals with MFD ˃ 30,000 mf/ml (Spearman’s correlation coefficient, *r*_s_ = 0.2, 95% CI − 0.3 to 0. 6, *P* = 0.486).

Tables 3 and 4 present the results for villages where at least two surveys were conducted, specifically focusing on data for individuals within the Bantu group (note that Pygmy individuals were also examined in the 1980s surveys). In 1985–1986, prevalences were calculated for the entire population (except for Panda), and it is estimated that if only adults were considered, the values would be scaled by a factor of 1.5–2. Thus, *L. loa* microfilaremia prevalence tended to decrease in four villages (Loyo, Mapati, Mayéyé and Mikakaya), remained relatively constant in Mambouana and Ouandzi, and saw a significant increase in Mbaya. In Panda, it is likely that the prevalence in the 1980s would have been < 27.7% if the microfilaremia evaluation had been performed on a thick smear, and it is probable that this prevalence increased at least slightly between that period and 2019. Table 5 illustrates the CMFL and MFD results obtained between the 2004 survey and the 2019 survey in seven villages, revealing no significant changes except for a notable decrease in the proportion of individuals with more than 30,000 mf/ml in Mikakaya. As for *M. perstans* microfilaremia, there was a striking decline in prevalence between the late 1980s and 2019, with intermediate values observed in 2004.

**Table 3:** Evolution of the prevalence of *Loa loa* microfilaremia in the villages of Lékoumou surveyed at least twice

Village	Examined 1985–1989^a^	Prev mf *Loa* 1985–1989 (%)	Examined 2004^b^	Prev mf *Loa* 2004 (%)	Examined 2019^c^	Prev mf *Loa* 2019 (%)	Statistical analysis
Loyo	433	24.5	68	23.5	248	17.3	*χ*^2^ = 1.353, *df* = 1, *P* = 0.245
Mambouana	341	13.8	84	25.0	71	21.1	*χ*^2^ = 0.328, *df* = 1, *P* = 0.567
Mapati	439	13.2	61	31.1	100	17.0	*χ*^2^ = 4.341, *df* = 1, *P* = 0.037^d^
Mayeyé			47	42.6	484	16.9	*χ*^2^ = 18.257, *df* = 1, *P* < 0.0001^d^
Mbaka			53	7.5	81	21.0	*χ*^2^ = 4.421, *df* = 1, *P* = 0.036^d^
Mikakaya			57	49.1	148	19.6	*χ*^2^ = 17.841, *df* = 1, *P* < 0.0001^d^
Ouandzi			54	25.9	87	24.1	*χ*^2^ = 0.058, *df* = 1, *P* = 0.809
Panda	101	27.7			72	29.2	*χ*^2^ = 0.047, *df* = 1, *P* = 0.829

^a^In Loyo, Mambouana and Mapati, subjects examined in 1985–1986 were ≥ 1 year, and two thick drops of 20 µl each were examined; in Panda, subjects were ≥ 20 years, and microfilariae were examined on 1 ml blood

^b^Subjects aged ≥ 15 years, mf tested on one 50-µl-thick drop

^c^Subjects aged ≥ 18 years, mf tested on a 50-µl-thick drop

^d^Statistically significant

**Table 4:** Evolution of the prevalence of *Mansonella perstans* microfilaremia in the villages of Lékoumou surveyed at least twice

Village	Examined 1985–1989^a^	Prev mf *Mp* 1985–1989 (%)	Examined 2004^b^	Prev mf *Mp* 2004 (%)	Examined 2019^c^	Prev mf *Mp* 2019 (%)	Statistical analysis
Loyo	432	22.2	68	8.8	248	1.2	Fisherʼs exact test, *P* = 0.004^d^, OR = 7.8, 95% CI 2–50
Mambouana	340	15.3	84	3.6	71	1.4	Fisherʼs exact test, *P* = 0.625, OR = 2.6, 95% CI 0–138
Mapati	438	19.2	61	3.3	100	1.0	Fisherʼs exact test, *P* = 0.558, OR = 3.3, 95% CI 0–200
Mayeyé			47	6.4	484	1.9	Fisherʼs exact test, *P* = 0.081, OR = 3.6, 95% CI 1–15
Mbaka			53	1.9	81	0	Chi-square test with Yates’s correction, *χ*^2^ = 0.046^d^, *df* = 1, *P* = 0.830
Mikakaya			57	1.8	148	0.7	Fisherʼs exact test, *P* = 0.480, OR = 2.6, 95% CI 0–207
Ouandzi			54	7.4	87	0	Chi-square test with Yates’s correction, *χ*^2^ = 4.218, *df* = 1, *P* = 0.040
Panda	101	5.9			72	0	Chi-square test with Yates’s correction, *χ*^2^ = 2.834, *df* = 1, *P* = 0.092

^a^In Loyo, Mambouana and Mapati, subjects examined in 1985–1986 were ≥ 1 year old, and two thick drops of 20 µl each were examined; in Panda, subjects were ≥ 20 years, and microfilariae were examined on 1 ml blood

^b^Subjects aged ≥ 15 years, mf tested on one 50-µl-thick drop

^c^Subjects aged ≥ 18 years, mf tested on a 50-µl-thick drop

^d^Statistically significant

**Table 5:** Trend in community microfilarial load (CMFL) and microfilarial densities of *Loa loa* microfilaremia in Lékoumou villages surveyed in 2004 and 2019

Village	CMFL *Loa* 2004^a^	CMFL *Loa* 2019^b^	Statistical analysis	% > 8000 mf/ml 2004^a^	% > 8000 mf/ml 2019^b^	Statistical analysis	% > 30,000 mf/ml 2004^a^	% > 30,000 mf/ml 2019^b^	Statistical analysis
Loyo	3.8	2.5	*t*-test, *t*(314) = 0.54, *P* = 0.587	5.9	4.8	Fisherʼs exact test, *P* = 0.756, OR = 1.2, 95% CI 0–4	0.0	1.6	Chi-square test with Yates’s correction, *χ*^2^ = 0.195, *df* = 1, *P* = 0.659
Mambouana	5.4	2.9	*t*-test, *t*(153) = 0.65, *P* = 0.516	4.8	0.0	Chi-square test with Yates’s correction, *χ*^2^ = 1.835, *df* = 1, *P* = 0.176	1.2	0.0	Chi-square test with Yates’s correction, *χ*^2^ = 0.001, *df* = 1, *P* < 0.001^c^
Mapati	6.8	2.4	*t*-test, *t*(159) = 1.40, *P* = 0.163	1.6	3.0	Fisherʼs exact test, *P* = 0.999, OR = 0.5, 95% CI 0–7	0.0	2.0	Chi-square test with Yates’s correction, *χ*^2^ = 0.143, *df* = 1, *P* = 0.705
Mayeyé	0.9	2.7	*t*-test, *t*(539) = − 0.65, *P* = 0.515	4.6	4.1	Fisherʼs exact test, *P* = 0.999, OR = 1.1, 95% CI 0–4	0.0	1.0	Chi-square test with Yates’s correction, *χ*^2^ = 0.001, *df* = 1, *P* = 0.999
Mbaka	1.0	4.9	*t*-test, *t*(132) = − 0.81, *P* = 0.418	1.9	9.9	Fisherʼs exact test, *P* = 0.087, OR = 0.2, 95% CI 0–1	0.0	1.2	Chi-square test with Yates’s correction, *χ*^2^ = 0.001, *df* = 1, *P* = 0.999
Mikakaya	3.7	3.1	*t*-test, *t*(203) = 0.21, *P* = 0.837	9.8	4.1	Fisherʼs exact test, *P* = 0.097, OR = 2.7, 95% CI 1–11	3.3	0.0	Chi-square test with Yates’s correction, *χ*^2^ = 0.247, *df* = 1, *P* = 0.619
Ouandzi	5.4	5.3	*t*-test, *t*(139) = 0.02, *P* = 0.983	5.6	8.0	Fisherʼs exact test, *P* = 0.741, OR = 0.7, 95% CI 0–3	0.0	2.3	Chi-square test with Yates’s correction, *χ*^2^ = 0.152, *df* = 1, *P* = 0.697

^a^Subjects aged ≥ 15 years old, mf tested on one 50-µl-thick drop

^b^Subjects aged ≥ 18 years, mf tested on a 50-µl-thick drop

^c^Statistically significant

## Discussion

A survey conducted as part of a screening initiative for a clinical trial evaluated the endemic levels of *L. loa* and *M. perstans* filariasis in 21 villages situated within a 30-km radius of the town of Sibiti, the capital of the Department of Lékoumou, Republic of Congo. ALthough our sampling relied on voluntary participation, the populations in each of these villages share highly homogeneous habits and ways of life, providing reassurance regarding the relative accuracy of our prevalence and infection intensity levels. Of these villages, six are positioned to the north of the town, while the remaining 15 are situated to the east on the two roads leading to the large village of Mayéyé. The vegetation in Lékoumou is predominantly dense forest, with areas along the roads characterized by forest degradation resulting from agricultural activities. Between Sibiti and Mayéyé, there are also expanses of savanna or bare ground [15].

The prevalence of *L. loa* microfilaremia within villages exhibited tendencies linked to village size and the surrounding forest cover type. Among the 21 surveyed villages, microfilaremia prevalences surpassing 20% were observed in 13, but slightly lower values of approximately 17% were recorded in three relatively populated areas: Loyo, Mapati and Mayéyé (2085, 1342 and 3647 inhabitants, respectively). This discrepancy is likely attributed to more extensive deforestation surrounding these larger villages, possibly resulting in lower *Chrysops* population density compared to the others, as illustrated by satellite images in Additional file 1: Fig. S2. Furthermore, the villages along the road from Sibiti to Mayéyé exhibited the lowest prevalences (2.8%, 10.6% and 14.5%), likely due to the particularly sparse vegetation cover in these areas. These findings are also influenced by the fact that *Chrysops* species rarely disperse beyond 2 km from sites favorable to them [16, 17].

The prevalence of *L. loa* microfilaremia demonstrated a significant sex disparity, with a higher prevalence in men (25.6%) compared to women (12.7%). These findings are consistent with those obtained in the same department in the 1980s [3], the southern region of Cameroon in 1995 [11] and several other studies [10, 13, 17, 18]. As previously suggested [10], this discrepancy may stem from unequal exposure to *Chrysops* bites during daily activities. Men, engaging more frequently in activities within the dense forest of the "Massif du Chaillu," spending extended periods away from home, are thus more exposed to repeated *Chrysops* bites than women. The proportion of individuals with high *L. loa* MFD (> 8000 mf/ml) was 5.1%, and those with very high MFD (> 30,000 mf/ml) constituted 1.1%. These findings align with those obtained in the Central Region of Cameroon [1]. In that context, the authors concluded that when the microfilaremia prevalence ranges between 20 and 30%, approximately 5–9% of adults exhibit a MFD > 8000 mf/ml and 1–3% of adults present a MFD > 30,000 mf/ml [19].

This study established a correlation between the prevalence of *L. loa* microfilaremia and three of the four indicators used to characterize the intensity of *L. loa* infection. Similar to findings in the Central Region of Cameroon [1], prevalence exhibited a significant correlation with CMFL, the arithmetic MFD mean and the proportion of individuals with a MFD > 8000 mf/ml. The lack of correlation between microfilaremia prevalence and the proportion of individuals with a MFD > 30,000 mf/ml could be attributed to the limited number of the latter (*n* = 28). Additionally, familial predisposition to *L. loa* hyper-microfilaremia contributed to clusters of individuals with this condition in specific villages, potentially resulting in a substantial number of individuals with very high MFD in villages with comparatively low prevalence [20]. The association between prevalence and intensity indicators of *L. loa* infection has a significant implication as it opens avenues for utilizing prevalence data to assess the level of risk associated with *L. loa* before implementing any public health measures. Furthermore, in alignment with the WHO roadmap for neglected tropical diseases [21], which recommends accelerating actions to control filariasis (onchocerciasis and lymphatic filariasis) in areas of coendemicity with loiasis, these results can aid in better guiding the planning and implementation of alternative treatment strategies against onchocerciasis in these villages [22].

This study compared the endemic levels measured in 2019 with those identified in previous surveys in eight villages. While several studies have evaluated the impact of mass ivermectin treatments on loiasis endemicity [21, 23], there is a dearth of documentation on the evolution of these indicators in areas that have not undergone any intervention. As far as we know, the only available data for assessing this spontaneous evolution are from studies conducted in 2001 and 2013 in five villages in the Eastern region of Cameroon (refer to Additional file 1: Table S1) [24].

In the Lékoumou region, a decline in microfilaremia prevalence was observed in four localities between the first survey and 2019. Notably, this reduction was significantly marked in Mayéyé (from 42.6 to 16.9% between 2004 and 2019) and Mikakaya (50.8% and 19.6% during the same period). In two villages (Mambouana and Ouandzi), the prevalence seemed to remain stable, while in two others (Panda, especially Mbaka), it appeared to be on the rise. While the relatively small number of participants in the 2004 surveys might contribute to some variability, it is unlikely to account for the more substantial decreases or increases. The rural exodus, particularly among men, following the May–October 1997 civil war in these villages could have influenced both population composition and infection levels. Additionally, pronounced deforestation around specific villages might explain a decrease in prevalence, whereas shifts in lifestyle or work location, such as an extended presence in the forest, could elucidate an increase in prevalence. Interestingly, despite potential significant variations in prevalence in some villages, intensity of infection (CMFL) remained stable.

The prevalence of *M. perstans* microfilaremia (1.0%) was notably lower than observed in other regions [1, 25, 26] and even lower than the rates documented in the Lékoumou region during the 1980s [4]. An entomological study conducted in April 1987 and January 1988 in a village in the Lékoumou region (Missama) indicated that over 98% of the *Culicoides* bites on humans were attributable to the species *Culicoides grahamii*. Captures were conducted daily from 7 to 9 a.m. and from 5 to 7 p.m., with an average number of bites per human per hour reaching 367 in April (midpoint of the "long rainy season") and 70.3 in January (start of the "short dry season") [26]. The decomposition of banana or plantain bunches is considered to be the preferred breeding ground for *C. grahamii* [27]. It is possible that the surface area of these environments has diminished over the last 30 years or that certain agricultural practices, notably the use of insecticides, have led to a substantial reduction in the population densities of *M. perstans* vectors.

## Conclusions

In 2019, the prevalence of *L. loa* microfilaremia in adults exceeded 20% in the majority (62%) of the surveyed villages. Previous parasitological survey data from 1985–1986 and 2004 revealed that in this region, which has not undergone mass ivermectin treatment for onchocerciasis control, the prevalence of *L. loa* infection has generally remained stable for 15 years or more. Nevertheless, variations in prevalence trends exist between villages, and alterations in the environment or in people's activity patterns could account for these differences. The results also indicate a dramatic decrease in the prevalence of *M. perstans* microfilaremia in the region﻿.

### Supplementary Information

**Additional file 1: Figure S1**: Distribution of *Mansonella perstans* cases by surveyed village in 2019. **Figure S2**: Satellite images and prevalence levels of *Loa loa* microfilaremia from each village in 2019. **Table S1**: Spontaneous evolution of *Loa loa* endemicity between 2001 and 2013 in five villages in Cameroon.

## Data Availability

Data supporting the conclusions of this article are included within the article. The datasets used and/or analyzed during the present study are available from the corresponding author upon reasonable request.

## References

[1] Boussinesq M, Gardon J (1997). Prevalences of *Loa loa* microfilaraemia throughout the area endemic for the infection. Ann Trop Med Parasitol.

[2] Zouré HGM, Wanji S, Noma M, Amazigo UV, Diggle PJ, Tekle AH (2011). The geographic distribution of *Loa loa* in Africa: results of large-scale implementation of the Rapid Assessment Procedure for Loiasis (RAPLOA). PLOS Negl Trop Dis.

[3] Noireau F, Apembet JD, Nzoulani A, Carme B (1990). Clinical manifestations of loiasis in an endemic area in the Congo. Trop Med Parasitol.

[4] Noireau F, Carme B, Apembet JD, Gouteux JP (1989). *Loa loa* and *Mansonella perstans* filariasis in the Chaillu mountains, Congo: parasitological prevalence. Trans R Soc Trop Med Hyg.

[5] Carme B, Ntsoumou Madzou V, Samba Y, Noireau F (1986). Prévalence des filarioses à microfilarémie au Congo. Bull OCEAC (Yaoundé).

[6] Hemilembolo MC, Niama AC, Campillo JT, Pion SD, Missamou F, Whittaker C (2023). Excess mortality associated with loiasis: confirmation by a new retrospective cohort study conducted in the Republic of Congo. Open Forum Infect Dis.

[7] Campillo JT, Bikita P, Hemilembolo M, Louya F, Missamou F, Pion SDS (2021). Safety and efficacy of levamisole in loiasis: a randomized, placebo-controlled, double-blind clinical trial. Clin Infect Dis.

[8] République du Congo. Annuaire Statistique: 2018. https://ins-congo.cg/annuaire-statistique-2018-Lékoumou/. Accessed 15 Jan 2023.

[9] Wilson EB (1927). Probable inference, the law of succession, and statistical inference. J Am Stat Assoc.

[10] Pion SDS, Gardon J, Kamgno J, Gardon-Wendel N, Chippaux JP, Boussinesq M (2004). Structure of the microfilarial reservoir of *Loa loa* in the human host and its implications for monitoring the programmes of community-directed treatment with ivermectin carried out in Africa. Parasitology.

[11] Mommers EC, Dekker HS, Richard P, Garica A, Chippaux JP (1995). Prevalence of *L. loa* and *M. perstans* filariasis in southern Cameroon. Trop Geogr Med.

[12] Gardon J, Gardon-Wendel N, Demanga-Ngangue, Kamgno J, Chippaux JP, Boussinesq M (1997). Serious reactions after mass treatment of onchocerciasis with ivermectin in an area endemic for *Loa loa* infection. Lancet.

[13] Pion SDS, Demanou M, Oudin B, Boussinesq M (2005). Loiasis: the individual factors associated with the presence of microfilaraemia. Ann Trop Med Parasitol.

[14] Tatuene JK, Fotsing RG, Nkoa T (2014). Epidemiology of *Loa loa* and *Mansonella perstans* filariasis in the Akonolinga health district, Centre Region, Cameroon. Health Sci Dis.

[15] Ministère de l'agriculture et de l'élevage, République du Congo. Monographie Lékoumou, secteur agricole. https://wri-sites.s3.amazonaws.com/forest-atlas.org/assets.forest-atlas.org/cog/resources/amenagementterritoire/Agriculture/Monographie_Lékoumou_final_SOFRECO-CERAPE.pdf. Accessed 20 Sept 2023.

[16] Duke BOL (1955). Studies on the biting habits of Chrysops. IV. The dispersal of Chrysops silacea over cleared areas from the rain-forest at Kumba, British Cameroons. Ann Trop Med Parasitol.

[17] Fischer P, Kilian D, Bamuhiiga J, Kipp W, Büttner D (1996). Prevalence of *Mansonella perstans* in western Uganda and its detection using the QBC-fluorescence method. Appl Parasitol.

[18] Thomson MC, Obsomer V, Kamgno J, Gardon J, Wanji S, Takougang I (2004). Mapping the distribution of *Loa loa* in Cameroon in support of the African Programme for Onchocerciasis Control. Filaria J.

[19] Boussinesq M, Gardon J, Kamgno J, Pion SDS, Gardon-Wendel N, Chippaux JP (2001). Relationships between the prevalence and intensity of *Loa loa* infection in the Central province of Cameroon. Ann Trop Med Parasitol.

[20] Eyebe S, Sabbagh A, Pion SD, Nana-Djeunga HC, Kamgno J, Boussinesq M (2018). Familial aggregation and heritability of *Loa loa* microfilaremia. Clin Infect Dis.

[21] Organisation mondiale de la Santé. 2021. Lutter contre les maladies tropicales négligées pour atteindre les objectifs de développement durable : feuille de route pour les maladies tropicales négligées 2021–2030. https://www.who.int/fr/publications/i/item/9789240010352. Accessed 20 Sept 2023.

[22] Kamgno J, Pion SD, Chesnais CB, Bakalar MH, D’Ambrosio MV, Mackenzie CD (2017). A test-and-not-treat strategy for onchocerciasis in *Loa loa*–endemic areas. N Engl J Med.

[23] Sumo L, Ntonifor NH, Afor AR, Bopda J, Bamou Heumou R, Ondoua Nganjou GS (2022). Loiasis is endemic in the Ndikinimeki Health District (Centre Region, Cameroon) but does not represent a hindrance to onchocerciasis elimination. Acta Trop.

[24] Takougang I, Meli J, Lamlenn S, Tatah PN, Ntep M (2007). Loiasis—a neglected and under estimated affliction: endemicity, morbidity and perceptions in eastern Cameroon. Ann Trop Med Parasitol.

[25] Asio SM, Simonsen PE, Onapa AW (2009). *Mansonella perstans* filariasis in Uganda: patterns of microfilaraemia and clinical manifestations in two endemic communities. Trans R Soc Trop Med Hyg.

[26] Noireau F, Itoua A, Carme B (1990). Epidemiology of *Mansonella perstans* filariasis in the forest region of South Congo. Ann Trop Med Parasitol.

[27] Hopkins CA (1952). Notes on the biology of certain *Culicoides* studied in the British Cameroons, West Africa, together with observations on their possible role as vectors of *Acanthocheilonema perstans*. Ann Trop Med Parasitol.

