# Supplementary material for: Loa loa and Mansonella perstans microfilaremia in the department of Lékoumou, Republic of Congo
Source: Parasit Vectors. 2023 Dec 9;16:451. doi: 10.1186/s13071-023-06056-w (PMC10710712; doi:10.1186/s13071-023-06056-w)
Supplement: Supplementary file 1 — Additional file 1: Figure S1: Distribution of Mansonella perstans cases by surveyed village in 2019. Figure S2: Satellite images and prevalence levels of Loa loa microfilaremia from each village in 2019. Table S1: Spontaneous evolution of Loa loa endemicity between 2001 and 2013 in five villages in Cameroon. [file 13071_2023_6056_MOESM1_ESM.docx]

**Additional file 1**

***Loa loa* and *Mansonella perstans* microfilaremia in the department of Lékoumou, Republic of Congo**

**Fig. S1.** Distribution of *M. perstans* cases by surveyed village in 2019


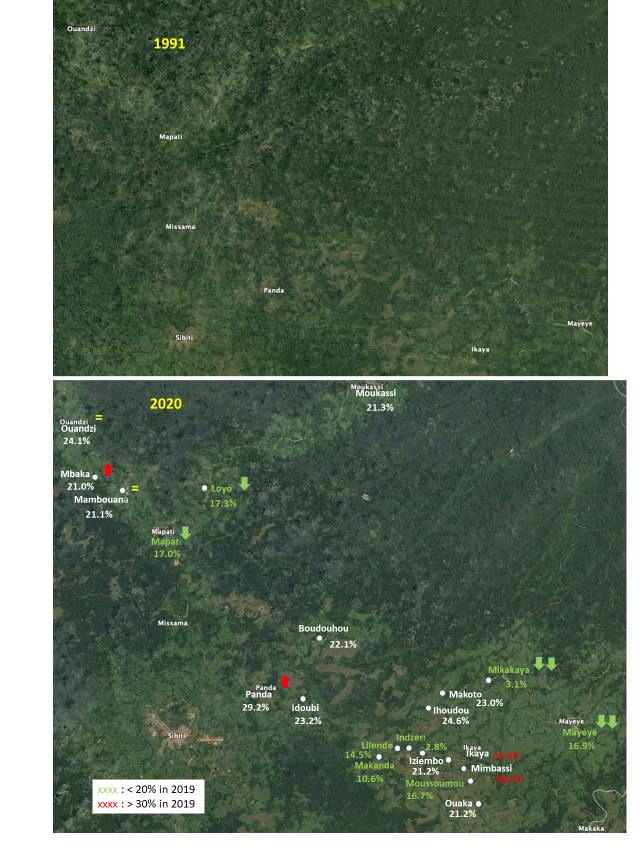


**Fig. S2.** Satellite images and prevalence levels of *L. loa* microfilaremia from each village in 2019.

Green and red arrows show villages where there was a decrease and an increase, respectively, in the prevalence of microfilaremia between the first surveys and the 2019 survey (see Table 3)

| Village | **Examined**  **Takougang data** | **Loa+ Takougang data** | **Prev *Loa* Takougang data** | **Examined**  **Takougang et al.** | ***Loa*+ Takougang et al.** | **Prev Loa Takougang et al.** | **Examined Wanji et al.** | ***Loa*+ Wanji et al.** | ***Prev* Loa Wanji et al** |
| --- | --- | --- | --- | --- | --- | --- | --- | --- | --- |
| **Screening year** | 2001 |  |  | 2001 |  |  | 2013 |  |  |
| **Age** |  |  | ≥ 15 ans |  |  |  | ≥ 10 ans |  |  |
| **Dem 2** | 99 | 37 | 37.4 | 99 | 37 | 37.4 | 40 | 13 | 32.5 |
| **Djal** | 83 | 22 | 26.5 | 86 | 26 | 30.2 | 44 | 14 | 31.8 |
| **Kamba Mieri** | 148 | 56 | 37.8 | 148 | 38 | 25.7 | 60 | 16 | 26.7 |
| **Konga** | 82 | 33 | 40.2 | 82 | 40 | 48.8 | 36 | 14 | 38.9 |
| **Ngoulmekong** | 25 | 9 | 36.0 |  |  |  | 52 | 21 | 40.4 |

**Table S1.** Spontaneous evolution of *L. loa* endemicity between 2001 and 2013 in 5 villages in Cameroon.
